# The Ghana heart initiative – a health system strengthening approach as index intervention model to solving Ghana’s cardiovascular disease burden

**DOI:** 10.3389/fpubh.2024.1330708

**Published:** 2024-04-17

**Authors:** Alfred K. Doku, John Tetteh, Juliette Edzeame, Ron J. G. Peters, Charles Agyemang, Elom Hillary Otchi, Alfred Edwin Yawson

**Affiliations:** ^1^University of Ghana Medical School, Accra, Ghana; ^2^Department of Public and Occupational Health, University of Amsterdam Medical Centre, University of Amsterdam, Amsterdam, Netherland; ^3^Department of Community Health, Medical School, University of Ghana, Accra, Ghana; ^4^Deutsche Gesellschaft fur Internationale Zusammenarbeit, Accra, Ghana; ^5^Department of Cardiology, Amsterdam University Medical Centers, University of Amsterdam, Amsterdam, Netherlands; ^6^Division of Endocrinology, Diabetes, and Metabolism, Department of Medicine, Johns Hopkins University School of Medicine, Baltimore, MD, United States; ^7^Accra College of Medicine, Accra, Ghana; ^8^Korle Bu Teaching Hospital, Accra, Ghana; ^9^Medical School, University of Ghana, Accra, Ghana

**Keywords:** cardiovascular disease, health system strengthening (HSS), case management guidelines, Akoma care app, Ghana heart initiative

## Abstract

Cardiovascular diseases (CVD) are the leading cause of death worldwide, with 80% of these deaths occurring in low-middle income countries (LMICs). In Ghana and across Africa, CVDs have emerged as the leading causes of death primarily due to undetected and under treated hypertension, yet less than 5% of resources allocated to health in these resource-poor countries go into non-communicable diseases (NCD) including CVD prevention and management. Consequently, most countries in Africa do not have contextually appropriate and sustainable health system framework to prevent, detect and manage CVD to achieve Universal Health Coverage (UHC) in CVD care through improved Primary Health Care (PHC) with the aim of achieving Sustainable Development Goals (SDG) in CVD/NCD. In view of this, the Ghana Heart Initiative (GHI) was envisaged as a national strategy to address the identified gaps using a health system and a population-based approach to reduce the national burden of CVDs. The GHI intervention includes the development of guidelines and training manuals; training, equipment support, establishment of a national call/support center, and improvement in the national data capturing system for CVDs and NCD, management of Hypertension, Deep Vein Thrombosis (DVT) and Heart Failure (HF). Following the implementation of the GHI concept, a national CVD Management Guideline was developed and 300-health facilities across the different levels of care including one teaching hospital, was also supported with basic life-saving equipment. In addition, more than 1,500 healthcare workers also reported improvement in their knowledge and skills in the management and treatment of CVD-related cases in their health facilities. These are key contributions to strengthening the health system for CVD care and learning lessons for scale up.

## 1 Problem description

The Global Burden of Disease Study indicates that ischemic heart disease and Stroke, both CVDs, were the leading causes of death in Ghana in 2017 (1). Hypertension has been described as the leading preventable cause of death worldwide and especially in lower-middle-income countries such as Ghana (2–4). Hypertension is the single most preventable risk factor for CVD in Ghana/Africa, primarily due to undetected, untreated and undertreated cases. In Ghana, population-based studies have shown an increase in hypertension prevalence reaching epidemic proportions and its significant impact on stroke morbidity and mortality, over the last four decades (5–7). Unfortunately, there is an inadequate level of awareness of hypertension and poor compliance to the therapy when diagnosed (6). In addition, up to 70% of persons identified with hypertension are not on treatment and only 13% of those with hypertension have their blood pressures adequately controlled (8). Nearly half of persons identified with hypertension have end organ damage suggesting that these persons have had long-standing disease without appropriate treatment (6). Risk factors such as low fruit and vegetable intake, low levels of physical activity, overweight/obesity, high blood pressure and raised cholesterol levels have been reported in Ghana (7–9). Some of these risk factors manifest as primordial risk conditions for hypertension and diabetes whilst cumulatively act as risk factors for target organ damage such as chronic kidney disease, heart diseases and stroke (7, 10).

Some efforts have been made to curb the rising CVD burden on the national level. In 2007, Ghana’s Ministry of Health (MOH) in its National Health Policy (NHP), announced a paradigm shift from curative to preventive services to empower communities to adopt healthy lifestyles (11). Similarly, in the revised NHP (2020), one of the its 5 policy objectives is to encourage the adoption of healthy styles. Some of the proposed core strategies include promotion of healthy eating, good nutrition and increased physical activity (12). The National Health Insurance Scheme (NHIS), the only government-subsidized health insurance for Ghanaian citizens, now caters for some medications for hypertension and diabetes on its essential drug list. In September 2011, the MOH participated in a United Nations (UN) High Level Meeting on NCDs and signed up to a ‘whole of society, whole of government’ approach to tackling the NCD crisis in LMICs. Ghana has also developed and launched the National Policy for the Prevention of Chronic NCDs 2019 and the Universal Health Coverage Roadmap 2020, all of which are aimed at prioritizing improvement in the outcomes of NCDs in Ghana as part of the national efforts toward attaining UHC.

These efforts notwithstanding, the increasingly devastating health and economic impact of CVDs, emphasizes the need to develop more practical, interventional and national model that can be aligned to existing models of healthcare to address this issue (1). The Ghana Heart Initiative (GHI) is a health system strengthening effort aimed at improving the prevention, detection and management of CVDs.

## 2 Available knowledge

The rising incidence of Non-Communicable Disease (NCDs) represents one of the greatest threats to health worldwide and has emerged as the leading cause of death globally. With an estimated 41 million deaths each year - equivalent to 71% of all deaths globally, reducing NCD mortality is recognized as a prime concern in the United Nations 2030 Agenda for Sustainable Development (1). The burden of NCDs is disproportionately felt in Low-and Middle-Income countries (LMIC) with about 80% affecting LMIC (2). Despite this burden of disease in LMIC, weak health systems with other challenges such as poor health financing, poor resources and low political commitment undermine efforts to effectively address the overwhelming NCD canker, prominent among which are the cardiovascular diseases (CVD) (13, 14).

Cardiovascular disease is the leading cause of death from NCDs globally (1), accounting for more than 50% of the global NCD burden, with the majority of CVD deaths occurring in LMICs (15). Sub-Saharan Africa (SSA) remained the only region of the world where CVD deaths increased between 1990 and 2013 (13). The CVD burden in SSA has been projected to double by 2030 if the status quo is maintained (13).

In Ghana, CVD-related deaths were the leading causes of deaths in 2021 according to the District Health Information Management System (DHIMS 2) of the Ghana Health Service (14). CVD deaths have been leading the chart in Ghana since 2017 driven mainly by uncontrolled hypertension. Ghana is currently experiencing a double burden of disease, with the increasing burden of NCDs especially CVDs as well as the interminable burden of infectious diseases (11). A national NCD policy was developed in 2011 and revised in 2022 to deal with this public health issue in a systematic fashion. However, the attainment of the policy goals and objectives was saddled with several challenges, leading to ineffective implementation (10).

## 3 Rationale

The burden of CVDs in Ghana remains high. Unfortunately, there have been limited nationally coordinated effort designed to improve the outcome of CVD care in Ghana. There are weaknesses in all the six WHO health systems building blocks with respect to CVD care in Ghana. An effective national governance system does not exist and the institutions tasked with the responsibility were weak and inactive. There was no national case management and treatment guideline for CVD care in Ghana prior to this intervention. This affected the extent of standardization in case management even in the same or similar health facilities. Moreso, most of the health facilities lacked basic life-saving equipment to provide quality healthcare to patients. An effective interventional approach to improve health may take on one of three tracks, namely: strengthening existing health systems, using population-based health approach or creating a parallel system to run along existing health structures. The GHI was envisaged as a national strategy to address the identified gaps using a health system and a population-based approach to reduce the national burden of CVDs.

### 3.1 Specific aims

The overall aim of the intervention was to improve risk assessment and management of CVDs at community as well as at the primary, secondary and tertiary levels of care in public health care facilities in Ghana. This was to be implemented within the health system framework or building blocks, to strengthen PHC and UHC in CVD care. Specifically, the intervention sought to:

- Develop CVD Case/Clinical Management Guidelines for all levels of care through the development of training manual and facilitator guide.
- Build the capacity of healthcare workers to effectively manage CVDs through implementation of training in general CVDs as well as basic and advance cardiac life support and deep vein thrombosis care.
- Provide life-saving equipment to implementing health facilities to effectively manage CVDs.
- Establish a CVD Support and Call Centers to compliment CVD case management and referral in Ghana;
- Develop hypertension management/reporting tools and improve the quality of CVD/NCD data in DHIMS 2 (District Health Information Management System).

## 4 Context

Ghana operates a pluralistic healthcare system comprising of public, private (i.e., self-financing and faith-based) and quasi health institutions. The Ministry of Health (MoH) is tasked with the responsibility of policy formulation and resource mobilization of the sector. The MoH has 26 Agencies with about 10 responsible for service delivery. The health system is also organized around a four-tiered system from national, regional, district and subdistrict levels, respectively, with the Ghana Health Service (GHS) being the largest public health care service delivery agency. There are five teaching hospitals (public) that provides tertiary healthcare service and also serve as the referral centers for secondary and primary health care facilities. The type of service provided by health facilities is defined by the credentialing of their health insurance level and accreditation by the Health Facilities Regulatory Agency (HeFRA). This notwithstanding, all public health facilities provide some CVD-related service either in the form of screening, health education and case management. The Non-Communicable Disease (NCD) outfit of the GHS is responsible for all public health related interventions of NCDs including CVDs in Ghana.

In a recent effort to address the existing gap in the approach to control the burden of CVDs in Ghana, a new intervention was launched called “The Ghana Heart Initiative (GHI).” The GHI project was a collaborative effort of the Ministry of Health (MOH)/ Ghana Health Service (GHS), Deutsche Gesellschaft fur Internationale Zusammenarbeit (GIZ) and Bayer, the AA program and other local and international stakeholders. It sought to strengthen the health system through capacity building in the areas of development and use of CVD-guidelines, training, equipment distribution, and establishment of a call center to facilitate referrals. It was a five-year project officially launched in January, 2019 but had been running from September 2018. The projects’ main objective was to improve outcomes of CVD by strengthening various components of the health system such as leadership & governance, human resource and health management information systems (HMIS).

Prior to this, the national CVD management had several limitations. For instance, there was variation in the management and treatment of CVD cases even within the same facility because of the non-existence of a national CVD Case Management Guideline. In addition, the knowledge, skills and competency of the health workforce in the management and treatment of CVD was suboptimal. Most of the health facilities did not also have the required equipment to be used for the management and treatment of CVDs; and the emergency and referral system in Ghana was also less developed.

Compounding these was the weak coordination or link between peripheral/lower-level health facilities which affects quality of patient care, leading to increased morbidities and mortalities across the country. CVDs present as either emergency *(acute stroke, myocardial infarction, pulmonary embolism or hemodynamically unstable arrhythmia)* or in a stable state. Unfortunately, the limited coordinated support especially to the peripheral/lower-level health facilities for CVD case management have contributed to poor case management and increased mortalities in Ghana and Africa.

The national governance structures for NCD and CVD was inactive and, in most instances, less functional. The quality of CVD-related data had challenges, CVD was generally underfunded in Ghana mainly attributable to limited governmental and international support. Data on CVD is essential to inform policy, planning, monitoring and evaluation. Unfortunately, there is inadequate quality data to project the real burden and the economic cost of CVDs in Ghana. Thus, the seeming neglect especially at the national level.

The GHI primarily sought to martial all efforts to strengthen the health system’s capacity toward improving the prevention, detection, diagnosis and management of CVDs in the communities as well as in the primary, secondary and tertiary levels of care in Ghana. With most Sub-Sahara African (SSA) countries facing similar health challenges, the successful implementation of GHI will be a model for other countries and similar settings to emulate.

## 5 Interventions

The interventions were implemented in a phased approach over the project implementation period, i.e., from 2018 to 2023 as a way of achieving the desired objectives.

**Pre-intervention phase:** This phase commenced in September 2018 and included baseline assessments of the equipment needs, assessment of the knowledge and competency of the health workforce and a health facility assessment for the management and treatment of CVD. This phase also included the purposive selection of 44 intervention health facilities across the entire level of the health system in the Greater Accra Region (GAR) of Ghana. There was also the establishment of a national governance committee comprising of members from the MoH, GHS, academia (teaching hospitals and universities in Ghana) and an international consultant representing GIZ and Bayer to oversee the successful implementation of the intervention.

## 6 Specific interventions

### 6.1 Development of the national CVD case management guidelines and training manuals/guides

Prior to the implementation of the GHI, there was no national CVD case management guideline in Ghana. This affected the standardization of case management and treatment since clinicians and health facilities were using their own preferred guidelines and protocols. In view of this, a National CVD Case Management Guideline was developed by a 100-member key stakeholders and expert group drawn from MOH, GHS, NCD Program (in the GHS), the Ghana Society of Cardiology, teaching and regional hospitals as well as the stakeholders from international cooperation and civil society groups across Ghana. The National Guideline for the Management of CVD was developed to provide a comprehensive CVD management protocol and to also serve as the foundational framework for the health system strengthening intervention by the GHI. The unique feature of the guideline is that, every healthcare provider involved in the management and treatment of CVD (i.e., doctor, physician assistant and nurse) at any level of the healthcare system (i.e., tertiary, secondary and primary) will be able to use it because of its adaptation to them. The main contents of the guideline include introduction to CVD, symptoms and signs of CVD, risk factors for CVD, cardiac arrest and the various diseases including hypertension, stroke, heart failure and arrhythmias among others. Further, in order to ensure that CVD training and capacity building was standardized and to ensure the availability of basic tools for the training of doctors, physician assistants and nurses across the country, training manuals and facilitators’ guides for CVD were developed from the National CVD Guideline. In order to ensure that the Guidelines were readily available and accessible to every provider and to ensure ease of reference and usage at the frontline, the “Akomacare App”- a mobile app that contained a softcopy of the Guideline was also developed and deployed. It created a platform for feedback or direct contact to the CVD Support/Call Centre. The name “Akoma” was derived from the local Ghanaian dialect meaning “heart.” The APP can be downloaded using PC, Android and IOS on the respective play stores.

### 6.2 Capacity building of healthcare workers to effectively manage CVD

The training and capacity building component of the project was meant to improve knowledge, skills and competencies of healthcare providers to change/influence the practice of CVD care in Ghana. There have been training and capacity building of 250 frontline healthcare providers of varied cadres/categories across the various levels of care in Basic and Advanced Cardiac Life Support (BLS/ACLS) and management of CVDs.

The BLS and ACLS training was conducted by Code Red, an AHA (American Heart Association) certified training center in Nigeria. Through this BLS/ACLS training by the GHI, an affiliate, Code Red Ghana has been created to run this training in Ghana and to ensure sustainability. Ten (10) AHA instructors have emerged in the Greater Accra Region with more instructors being groomed as the training is rolled out nationwide.

A total of 76 trainer-of-trainers and over 1,500 health workers have also been trained in various competencies in CVD management. The training included many practical sessions in blood pressure and Electrocardiogram (ECG) measurement and interpretation of findings, simulations, and resuscitations.

### 6.3 Equipment support

Informed by the health facilities baseline and equipment needs assessment that were undertaken in the 44 implementing facilities in the GAR, various CVD management and diagnostic equipment have been supplied. These include:

- Diagnostic tools: weighing scales, height meters, glucometers, International Normalized Ratio (INR) devices, N-Terminal-proB-type Natriuretic Peptides (NT-pro-BNP) devices, sphygmomanometers, ECG machines, compression ultrasound machines and echocardiogram machines.
- Management tools: patient monitors and biphasic manual defibrillators.

It is estimated that, over 300 facilities across the country also received these set of equipment when the project was finally rolled out to 10 other regions.

### 6.4 National CVD support/call center

A national CVD Support/Call Center was established and situated in the Korle Bu Teaching Hospital (KBTH) as a way of ensuring effective coordination and management of CVD cases at the peripheries. The primary aim of the call center was to provide topside support: build capacity for onsite management of cases rather than referral to higher levels because of challenges with referrals such as ‘no bed syndrome’, weak ambulance system, higher cost of care etc. The Center receives and directs calls to identified Cardiologists and Neurologists in the Hospital who provide guidance and support in the management of CVD cases. The intent was to empower health professionals at the peripheries to be able to manage CVD cases there other than quickly referring and clogging up the secondary and tertiary levels. The Center however facilitates referrals of cases in the event that the case is determined to be above the capacity of the periphery. The staff of the Call Center is multidisciplinary with nurses who are the first responders of the call and then subsequently direct to the appropriate specialists and/or consultants.

### 6.5 Development of hypertension management E-Tracker tool and CVD data quality improvement in the DHIMS2

The GHI partnered with PATH, NCD Program and PPME of the GHS to improve the quality of NCD data in Ghana. Most of these data sets had CVDs as well. This has led to an increase in the current indicators or variables from 18 to 66 to allow for more disaggregated data. For instance, hypertension was only reported as hypertension, but the modification has included indicators/variables such as newly diagnosed hypertension, those on treatment, those compliant on treatment, those controlled and those with complications (target organ damage: TOD) such as chronic kidney diseases, hypertensive heart diseases and stroke. Similarly, diabetes, cancer and other diseases have been expanded accordingly and consistent with global best practice. In addition to improvement in monthly outpatient data set, new ones have been created including screening, wellness clinic, referral and consulting room register for CVDs.

A novel hypertension management tool was developed from the E-Tracker, an existing management tool for HIV and Tuberculosis and has been adapted for use in the management of NCD with hypertension as an index disease. The E-Tracker for Hypertension is used for community hypertension screening and evaluation. It included management and monitoring at the health facility level as well as management and monitoring in the community and the community pharmacy/chemical sellers.

### 6.6 Management of specific risk factors/CVDs

There are other components of the GHI project that looked at improving other CVD-related conditions such as heart failure (HF) and deep vein thrombosis (DVT). The HF Management component sought to establish nine heart failure centers of excellence in five teaching hospitals, two regional and two district hospitals, respectively, and provide training. Equipment such as point of care echocardiogram machines and Pro-BNP devices were supplied to these health facilities. A national heart failure registry, which migrated into a national CVD registry was used to capture data and to monitor/evaluate care. A number of efforts have also been made to improve outcomes of DVT patients including establishment of a National DVT Training Center in the Komfo Anokye Teaching Hospital (KATH) in Kumasi and a subsequent upscaling to eight other beneficiary facilities. The DVT intervention involved development of advanced DVT management and training manual, training in DVT management, a point of care compression ultrasound, INR and D-Dimer devices support. In all, 36 health personnel benefited from the advanced training and records of improvement in the detection and management of over 1,300 participants.

### 6.7 Institutionalization of CVD training

Efforts have been made to ensure that CVD training in health training institutions is improved especially for doctors and nurses. The first stakeholder meeting was held in February 2021 with stakeholders and partners from University of Ghana Medical School, University of Health and Allied Health, Nursing and Midwifery Council, Ghana College of Physician and Surgeons, and the NCD Program of the Ghana Health Service (GHS). In addition, discussions are also ongoing with the Institutional Care Division (ICD) of the GHS and Faith-Based Organizations (FBOs) to make training in CVDs a part of their in-service training and as one of their Key Performance Indicators (KPIs). The health professions regulatory councils of doctors and nurses have also been engaged to make CVD in-service training a CPD/CME accredited; and to make BLS/ACLS a requirement of recertification by these professionals every 2 years. The development of e-learning platforms for CVD are also underway to make the programs more accessible to the targeted cadre of health workers and the gains, sustainable.

## 7 Conclusion

The GHI represents a concerted effort at reducing the morbidity and mortality from CVDs in Ghana by a multifaceted systematic approach. This is to ensure that NCDs, particularly CVDs, is tackled holistically and integrated in the existing health system, to serve as a basis for a unified sustainable approach to CVD risk assessment and management in Ghana and to serve as a model to countries with similar low to middle-income settings or health systems to emulate.

## Data availability statement

The original contributions presented in the study are included in the article/supplementary material, further inquiries can be directed to the corresponding author/s.

## Author contributions

AD: Conceptualization, Validation, Writing – original draft. JT: Formal analysis, Methodology, Writing – review & editing. JE: Resources, Supervision, Writing – review & editing. RP: Writing – review & editing. CA: Writing – review & editing, Supervision. EO: Methodology, Supervision, Writing – original draft, Writing – review & editing. AY: Supervision, Writing – review & editing.
